# Disparities in Plasma Homocysteine Levels Between Early-Onset and Late-Onset Depression

**DOI:** 10.1155/2024/7919736

**Published:** 2024-09-20

**Authors:** Tianle Wang, Qiang Wang, Huarong Zhou, Xiaomei Zhong, Ying-Chun Dai, Jiubo Zhao, Zezhi Li, Yuping Ning

**Affiliations:** ^1^The Affiliated Brain Hospital, Guangzhou Medical University, Guangzhou, China; ^2^Key Laboratory of Neurogenetics and Channelopathies of Guangdong Province and the Ministry of Education of China, Guangzhou Medical University, Guangzhou, China; ^3^Guangdong Engineering Technology Research Center for Translational Medicine of Mental Disorders, Guangzhou, China; ^4^Department of Epidemiology, School of Public Health, Southern Medical University (Guangdong Provincial Key Laboratory of Tropical Disease Research), Guangzhou, China; ^5^Department of Psychology, School of Public Health, Southern Medical University, Guangzhou, China; ^6^Department of Psychiatry, Zhujiang Hospital, Southern Medical University, Guangzhou, China

**Keywords:** cognition, early-onset, homocysteine, late-life depression, memory

## Abstract

**Background:** Elevated homocysteine levels and late-life depression are risk factors for cognitive decline: a comparative study highlighted the association of late-onset depression (LOD) with more significant cognitive deficits and brain pathology than early-onset depression (EOD). Limited research has explored the possible interaction between homocysteine levels and their correlation with cognitive performance in patients with EOD and LOD.

**Methods:** Fifty-seven individuals with EOD, 56 with LOD, and 89 matched healthy controls (HC) were recruited. Global cognition, memory, execution, language, attention, visuospatial skills, and plasma homocysteine levels were examined.

**Results:** Compared with HC and patients with EOD, patients with LOD had higher plasma homocysteine levels (*p* < 0.05), with no significant difference between HC and patients with EOD (*p* > 0.05). Furthermore, homocysteine levels and diagnosis groups showed significant main effects on depression and cognition, with no significant interaction effects being observed. Additionally, plasma homocysteine levels were negatively correlated with global cognition, attention, visuospatial skills, and executive function in patients with LOD (*p* < 0.05).

**Conclusions:** Compared with HC and patients with EOD, elevated homocysteine levels in patients with LOD were independently associated with cognitive performance. The potential therapeutic efficacy of homocysteine-lowering B-vitamin supplementation could be explored as a viable intervention to mitigate the documented debilitating effects of cognitive deficits in this population.

## 1. Introduction

Late-life depression (LLD) is a pervasive and disabling mental disorder, referring to a major depressive disorder occurring in individuals aged 60 years and older. The prevalence of LLD ranges from 4.6% to 9.3% [1], with more than half of affected individuals (52%) exhibiting mild cognitive impairment [2]. The association between depression and cognitive impairment may stem from distinct mechanisms in early-onset depression (EOD), characterized by the first episode occurring before the age of 60, and late-onset depression (LOD), characterized by the first episode occurring after the age of 60. Hypercortisolemia is implicated in linking cognitive impairment to EOD, while vascular risk and white matter (WM) hyperintensity are considered risk factors for LOD [3]. Longitudinal studies have revealed that patients with LOD, but not those with EOD, are at a greater likelihood of developing dementia and an increased risk of all-cause mortality [4].

Homocysteine serves as a risk factor not only for stroke, cerebral vasculature, and vascular dementia but also for degenerative dementias [5]. A recent statement [6] proposed that homocysteine is a modifiable risk factor for cognitive impairment and dementia. Studies suggest that the relative risk of dementia in older adults with elevated homocysteine levels ranges from 1.15 to 2.5, with the population-attributable risk ranging from 4.3% to 31% [6]. Furthermore, clinical studies have demonstrated that homocysteine-lowering therapy with B vitamins significantly improves cognitive function in patients with mild cognitive impairment [7].

Our previous studies established that patients with LLD exhibit elevated plasma homocysteine levels, more pronounced cognitive impairment, and increased WM damage compared to elderly individuals without LLD [8]. Longitudinal investigations have suggested that deficiencies in folate and vitamin B (12), as well as elevated homocysteine levels, may contribute to the risk of LLD [9]. Although the association among LLD, homocysteine levels, and cognitive impairment are well recognized, investigations of plasma homocysteine levels in LOD patients, which are closely associated with cognitive impairment, remain limited. Furthermore, given the differences in onset age between the EOD and LOD subtypes, it remains uncertain whether they are distinctly related to cognitive impairment and whether elevated plasma homocysteine is correlated with specific cognitive domain impairments in LOD.

This study aimed to investigate changes in plasma homocysteine levels and their associations with cognitive function in normal elderly individuals and those with EOD and LOD patients. We hypothesized that (1) plasma homocysteine levels would be greater in LOD patients than in both normal elderly individuals and the patients with EOD and (2) elevated homocysteine levels are linked to cognitive impairment, particularly in memory function, in LOD patients.

## 2. Methods

### 2.1. Participants

A total of 57 participants who were diagnosed with EOD and 56 with LOD were enrolled from the Affiliated Brain Hospital of Guangzhou Medical University, along with 89 age-matched healthy controls (HC) recruited from communities in Guangzhou. All participants or their legal guardians provided informed consent before participating in the study. The Ethics Committees of the Affiliated Brain Hospital of Guangzhou Medical University granted approval for the present study (no. 2014-016).

The inclusion criteria for LLD patients were as follows: (1) aged ≥60 years and (2) diagnosed with a major depressive disorder based on the criteria outlined in the Diagnostic and Statistical Manual of Mental Disorders, Fourth Edition. The exclusion criteria, as previously detailed [10], included (1) a history of other major mental illnesses; (2) a history of schizophrenia or bipolar disorder; (3) recent transcranial magnetic stimulation or electroconvulsive treatment within the past month; (4) neurological disorders, such as brain tumor and stroke; and (5) physical disorders, such as hypothyroidism and anemia, known to induce emotional disturbances. HC individuals were cognitively intact individuals without a history of depression.

### 2.2. Neuropsychological Assessments

An experienced psychologist performed the standardized cognitive evaluation. Global cognition was measured using the mini-mental state examination (MMSE) [11]. Fiveneuropsychological tests were used to evaluate the following cognitive domain functions: (1) memory function, evaluated by the Rey–Osterrieth complex figure (ROCF)-delay recall test [12] and auditory verbal learning test (AVLT) [13], (2) executive function, tested by the time of Part B of the trail-making test (TMT-B) [14] and (Stroop color word test [SCWT])-C [15], (3) language function, evaluated by the verbal fluency test (VFT) [16] and Boston naming test (BNT) [17], (4) attention function, tested by the symbol digit modalities test (SDMT) [18] and TMT-A [14], and (5) visuospatial skill, assessed by the clock drawing test 4 (CDT4) [19] and ROCF [12]. Depressive symptoms were measured using the 17-item Hamilton depression rating scale (HAMD-17).

### 2.3. Plasma Homocysteine Concentration Measurements

Previous studies have detailed the homocysteine measurement procedure [10]. Plasma homocysteine concentrations were assessed employing an enzyme cycling assay conducted by automated analyzers (AU5800 testers, Beckman Coulter, Brea, CA). All samples were analyzed by a research assistant blinded to the subjects' status. Participants with homocysteine values ≥12.9 µmol/l, which corresponds to the two-thirds point in the dataset, were categorized into the high homocysteine (HighHCY) group. In comparison, those with homocysteine values <12.9 µmol/l were classified into the low homocysteine (LowHCY) group.

### 2.4. Statistical Analysis

The data were analyzed using the Statistical Package for the Social Sciences version 25.0 (SPSS 25.0) and the R statistical software (v 3.7.0). The Kolmogorov‒Smirnov test was used to detect the normality of the variable distribution. Two-tailed chi-square tests were employed for between-group analysis of gender composition. One-way analysis of variance (ANOVA) was utilized to compare plasma homocysteine levels, years of education, age at the onset of the first depressive episode, duration of depression, and neuropsychological assessments across two or three groups. Post hoc least significant difference tests were applied for multiple comparisons in the one-way ANOVA. For the difference in homocysteine levels between HC, the patients with EOD, and the patients with LOD, 2 × 3 ANCOVA (homocysteine levels × diagnosis group) was performed to control confounding factors, with each depression and cognition index as the dependent variable and homocysteine levels and diagnosis group as fixed factors. The main effects of homocysteine level, diagnosis group, and homocysteine level × diagnosis group interaction were determined in each model. A partial correlation analysis was performed to investigate the association between homocysteine levels and cognitive characteristics in HC, patients with EOD, and patients with LOD, controlling for age, sex, years of education, HAMD-17 scores, age at first depressive episode, and duration of depression.

For result robustness, we conducted additional nonparametric tests using the Kruskal‒Wallis test for three-group comparisons. General linear models were used to assess the main effects of homocysteine level, diagnosis group, and their interaction. Spearman correlation analysis of associations between cognition and plasma homocysteine levels among the HC, EOD, and LOD groups.

## 3. Results

### 3.1. Demographic Data, Cognitive Function, and Plasma Homocysteine Levels

The demographic data for the various groups are presented in Table 1. Participants in the LOD group were older and had elevated plasma homocysteine levels compared to those in the HC and EOD groups (*p* < 0.05), with no significant differences observed between the HC and EOD groups. Patients in the EOD group exhibited a longer duration of depression than did those in the LOD group. Both the EOD and LOD groups exhibited poorer global cognition, memory, attention, and executive function than did the HC group (*p* < 0.05), with no significant difference observed between the EOD and LOD groups (*p* > 0.05) (Table 1).

Due to the nonnormal distributions of certain variables, including variables such as age at first depressive episode onset, duration of depression, HAMD-17 score, homocysteine levels, MMSE score, ROCF scores (including delay recall), SCWT-C time, TMT-B time, TMT-A time, and CDT score, we used the Kruskal‒Wallis test for three-group comparison. The main results from these nonparametric tests were consistent with those from the parametric tests (Table S1).

### 3.2. Depression and Cognitive Performance Associated With Different Homocysteine Levels

Table 2 shows the distributions of participants across groups based on homocysteine levels. In the HC group, 64 participants had low levels, and 25 had elevated levels. For the EOD group, 42 participants had low levels, while 15 had elevated levels. The LOD group included 25 participants with low levels and 31 with elevated levels.

As shown in Table 2, 2 × 3 ANCOVA (homocysteine levels × diagnosis group) demonstrated that homocysteine levels exhibited significant main effects on the age at the first depressive episode, global cognition, memory, attention, and language function (all *p* values after Bonferroni <0.05), without significant impact on the duration of depression and depression (HAMD) (all *p* values after Bonferroni > 0.05). The diagnosis group exhibited significant main effects on depression and cognitive function (all *p* values after Bonferroni <0.05), except for the age at the first depressive episode (*p*=0.408), which was not significantly affected. However, the homocysteine levels and diagnosis group had no significant interaction effect on depression or cognitive function (all *p* values > 0.05).

To ensure result stability, the Mann‒Whitney *U* test and a general linear model were used to confirm the results of the 2 × 3 ANOVA. The main results from these nonparametric tests were consistent with those from the parametric tests (Tables S2 and S3). Interestingly, the general linear model further revealed that homocysteine level and diagnosis group had an interaction effect on language function (BNT scores) (*p*=0.025, uncorrected).

### 3.3. Relationships Between Cognition and Plasma Homocysteine Levels

Table 3 summarizes the significant associations between plasma homocysteine levels and neuropsychological scale scores. In the LOD group, global cognition (MMSE scores), executive function (1/SCWT-C time), attention (SMDT scores), and visuospatial skills (ROCF and CDT scores) were negatively correlated with plasma homocysteine levels (*p* < 0.05). Conversely, no significant associations existed between cognition and plasma homocysteine levels in the HC and EOD groups. Additionally, Spearman correlation analysis revealed that only global cognition (MMSE scores) exhibited a negative correlation with plasma homocysteine levels in the LOD group (Table S4).

## 4. Discussion

This study is the first investigation to investigate changes in plasma homocysteine levels and their relationships with depression and cognition in healthy elderly individuals, the patients with EOD, and the patients with LOD. The key findings were as follows: First, patients with LOD exhibited greater plasma homocysteine levels than healthy elderly individuals and EOD patients (*p* < 0.05), with no significant difference between healthy elderly individuals and EOD patients (*p* > 0.05). Second, the homocysteine levels and diagnosis groups showed significant main effects on depression and cognition, with no significant interaction effects observed. Third, plasma homocysteine levels were negatively correlated with global cognition, attention, visuospatial skills, and executive function in patients with LOD (*p* < 0.05).

As expected, patients with LOD exhibited increased plasma homocysteine levels compared to HC and the patients with EOD, suggesting an increased risk of cerebrovascular diseases or degenerative dementias. This finding aligns with previous research demonstrating that elevated homocysteine is a recognized risk factor for cerebrovascular diseases, stroke, and vascular dementia [20]. Furthermore, compared with EOD, LOD is more closely associated with vascular risk factors and WM hyperintensity [21]. Previous research has also demonstrated that the interplay between elevated homocysteine levels and WM abnormalities exacerbates cognitive impairment severity [10]. Additionally, elevated homocysteine has been identified as an independent risk factor for Alzheimer's disease (AD) [22] and is linked to faster cognitive deterioration[23]. A positive correlation between homocysteine and A*β* 1-40 deposition in the brains of AD patients has been reported [24]. Homocysteine is thought to induce or potentiate the intracellular and extracellular accumulation of A*β* 1-42 [25] and seems to be involved in tau protein metabolism [26]. Our study further supports the notion that LOD patients are at an elevated risk of both vascular diseases and progressive degenerative dementia.

This study revealed a correlation between plasma homocysteine levels and global cognitive function in LOD patients but not in healthy elderly individuals or EOD patients. Previous reviews have reported that plasma homocysteine concentrations are greater in patients with moderate-to-severe dementia than in those with mild cognitive impairment and dementia [27]. The present study suggested that elevated plasma homocysteine levels are associated with cognitive impairment in LOD patients. The anatomic-functional relevance of homocysteine may help to elucidate the mechanisms by which homocysteine is associated with cognitive impairment. Some studies have shown that subjects with higher homocysteine levels exhibit atrophy in the frontal, parietal, and temporal cortex [28], WM damage [8], and hippocampal atrophy [29]. Our findings confirm the strong association between homocysteine levels and cognitive function, suggesting that homocysteine levels may indicate cognitive performance in LOD patients.

Furthermore, the association between plasma homocysteine levels and cognitive impairment, particularly in the attention domain (SMDT score), was solely observed in the LOD group and not in the EOD group, potentially due to the small sample size. Although EOD patients exhibited a similar trend to LOD, the *p*-value did not reach <0.05 (e.g., *p*=0.058 for the correlation between homocysteine and SMDT score). Previous studies have not extensively explored the impact of homocysteine on each domain of cognitive function in LOD patients. Our study supports the findings of [30, 31], revealing negative correlations between homocysteine levels and attention span in older people. This finding underscores the potential therapeutic value of homocysteine-reduced B-vitamin supplementation, particularly in addressing cognitive deficits related to attention domains in LOD patients.

The intricate relationship between plasma homocysteine levels and depression was also investigated. Our previous study indicated that elevated homocysteine levels and LLD were associated with worse cognitive performance [10]. Furthermore, longitudinal studies have shown that higher baseline homocysteine levels are associated with an increased risk of incident depression at follow-up [9]. This complex interaction may be explained by the role of homocysteine as an endogenous agonist of the N-methyl-D-aspartate (NMDA) receptor, a component of the glutamate receptor system. Disruptions in glutaminergic transmission are known to occur in depression, and NMDA antagonists are considered potential therapeutic options for depression and cognitive deficits [32]. However, further exploration is warranted to fully understand the relationships among elevated homocysteine levels, depressive symptoms, and cognitive function.

## 5. Limitations

The current study has several limitations. First, the findings are based on a cross-sectional analysis, and there is no direct evidence regarding cerebrovascular disease or neurodegeneration in LOD patients. Follow-up studies are essential to validate whether elevated homocysteine levels can predict the development of cerebrovascular disease or cognitive dysfunction in individuals with LOD. Second, the study did not assess factors influencing homocysteine levels, such as diet, physical activity, and lifestyle. Future research will incorporate an examination of these factors. Third, the present study did not exclude the potential influence of medication on LLD patients, which may have impacted the results. Fourth, the limited size of the EOD group in this study may have influenced the lack of association between plasma homocysteine levels and cognitive impairment. Future research with larger samples and longitudinal designs will clarify plasma homocysteine level discrepancies between EOD and LOD.

## 6. Conclusion

In conclusion, patients with LOD exhibit elevated plasma homocysteine levels, which are linked to cognitive impairment. Plasma homocysteine levels may be a straightforward and cost-effective biomarker for predicting cerebrovascular disease or neurodegeneration in LOD patients. The use of homocysteine-reduced B-vitamin supplementation could be explored as a potential therapeutic strategy to alleviate the disabling effects of cognitive deficits in patients with LOD.

## Figures and Tables

**Table 1:** Demographic data, cognitive function, and plasma homocysteine levels of participants.

Variables	HC *N* = 89	EOD *N* = 57	LOD *N* = 56	*p*.Overall	*p*.HC vs. EOD	*p*.HC vs. LOD	*p*.EOD vs. LOD
Homocysteine status:	—	—	—	<0.001⁣^*∗∗*^	0.965	0.005⁣^*∗∗*^	0.005⁣^*∗∗*^
Low homocysteine	64 (71.9%)	42 (73.7%)	25 (44.6%)	—	—	—	—
High homocysteine	25 (28.1%)	15 (26.3%)	31 (55.4%)	—	—	—	—
Gender:	—	—	—	0.286	0.364	1.000	0.364
Male	25 (28.1%)	10 (17.5%)	16 (28.6%)	—	—	—	—
Female	64 (71.9%)	47 (82.5%)	40 (71.4%)	—	—	—	—
Age (years)	66.5 (5.60)	64.7 (5.12)	70.4 (6.73)	<0.001⁣^*∗∗*^	0.182	<0.001⁣^*∗∗*^	<0.001⁣^*∗∗*^
Years of education	9.90 (2.20)	9.16 (2.63)	9.73 (3.48)	0.269	0.247	0.932	0.504
Age at first depressive episode (years)	—	49.0 (10.1)	67.7 (6.37)	<0.001⁣^*∗∗*^	—	—	<0.001⁣^*∗∗*^
Duration of depression (years)	—	10.2 (9.94)	2.87 (3.06)	<0.001⁣^*∗∗*^	—	—	<0.001⁣^*∗∗*^
HAMD	1.46 (2.25)	10.2 (8.48)	11.1 (8.36)	<0.001⁣^*∗∗*^	<0.001⁣^*∗∗*^	<0.001⁣^*∗∗*^	0.715
Homocysteine (μmol/l)	11.5 (2.92)	11.7 (3.40)	14.1 (5.90)	<0.001⁣^*∗∗*^	0.949	<0.001⁣^*∗∗*^	0.004⁣^*∗*^
Global cognition							
MMSE	27.8 (1.16)	24.7 (3.61)	23.9 (4.04)	<0.001⁣^*∗∗*^	<0.001⁣^*∗∗*^	<0.001⁣^*∗∗*^	0.334
Memory							
AVLT N1-N5 total	33.5 (7.96)	26.1 (11.9)	26.1 (9.54)	<0.001⁣^*∗∗*^	<0.001⁣^*∗∗*^	<0.001⁣^*∗∗*^	0.999
ROCF-delay recall	11.8 (6.22)	7.63 (6.05)	7.99 (6.05)	<0.001⁣^*∗∗*^	<0.001⁣^*∗∗*^	0.002⁣^*∗*^	0.952
Executive function							
SCWT-C time	45.5 (4.18)	43.4 (5.23)	43.8 (5.51)	0.024	0.036⁣^*∗*^	0.109	0.946
TMT-B time	62.2 (26.0)	77.6 (28.0)	85.1 (27.7)	<0.001⁣^*∗∗*^	0.003⁣^*∗∗*^	<0.001	0.340
Language							
BNT	22.0 (2.95)	19.4 (3.87)	20.3 (3.65)	<0.001⁣^*∗∗*^	<0.001⁣^*∗∗*^	0.013	0.416
VFT	15.3 (3.84)	13.2 (3.88)	13.8 (4.18)	0.005⁣^*∗∗*^	0.006⁣^*∗∗*^	0.074	0.743
Attention							
SDMT	35.7 (9.32)	28.2 (9.65)	25.3 (11.8)	<0.001⁣^*∗∗*^	<0.001⁣^*∗∗*^	<0.001⁣^*∗∗*^	0.331
TMT-A time	50.3 (18.3)	63.1 (25.0)	68.6 (26.6)	<0.001⁣^*∗∗*^	0.003⁣^*∗∗*^	<0.001⁣^*∗∗*^	0.427
Visuospatial skill							
ROCF	27.3 (4.74)	23.8 (6.70)	24.7 (5.97)	0.001⁣^*∗∗*^	0.001⁣^*∗∗*^	0.027⁣^*∗*^	0.704
CDT	3.74 (0.55)	3.39 (0.82)	3.36 (0.83)	0.002⁣^*∗∗*^	0.013⁣^*∗*^	0.008⁣^*∗∗*^	0.970

*Note:* The continuous variables in the table are expressed as the mean and standard deviation.

Abbreviations: AVLT, auditory verbal learning test; BNT, Boston naming test; CDT, clock drawing test; EOD, early-onset depression; HAMD-17, Hamilton depression rating scale, 17 items; HC, healthy controls; LOD, late-onset depression; MMSE, mini-mental state examination; ROCF, Rey‒Osterrieth complex figure; SCWT, Stroop color word test; SDMT, symbol digit modalities test; TMT, trail making test; VFT, verbal fluency test.

⁣^*∗*^*p* < 0.05, ⁣^*∗∗*^*p* < 0.01.

**Table 2:** Depression and cognitive performance associated with different homocysteine levels (covariant: age).

Variables	HC		EOD		LOD		Homocysteine status *F* (*p*)	Group status *F* (*p*)	Homocysteine × Group status *F* (*p*)
	LowHCY *N* = 64	HighHCY *N* = 25	LowHCY *N* = 42	HighHCY *N* = 15	LowHCY *N* = 25	HighHCY *N* = 31			
Age of first depressive episode (years)	—	—	48.9 (10.3)	49.2 (10.1)	66.4 (5.58)	68.7 (6.85)	139.818 (<0.001⁣^*∗∗*^)	0.690 (0.408)	0.191 (0.663)
Duration of depression (years)	—	—	11.2 (10.5)	7.33 (7.73)	3.27 (2.87)	2.54 (3.22)	2.409 (0.124)	32.385 (<0.001⁣^*∗∗*^)	0.647 (0.423)
HAMD	1.53 (2.35)	1.28 (2.01)	10.0 (8.74)	10.7 (7.96)	10.2 (8.79)	11.9 (8.06)	0.402 (0.527)	49.988 (<0.001⁣^*∗∗*^)	0.312 (0.732)
Global cognition									
MMSE	27.9 (1.19)	27.7 (1.11)	25.2 (3.12)	23.5 (4.63)	25.1 (3.75)	23.0 (4.09)	7.19 (0.007⁣^*∗∗*^)	36.93 (<0.001⁣^*∗∗*^)	2.06 (0.13)
Memory									
AVLT N1–N5 total	34.4 (7.55)	31.2 (8.67)	27.7 (12.1)	21.7 (10.3)	28.1 (9.57)	24.3 (9.32)	8.106 (0.005⁣^*∗∗*^)	15.326 (<0.001⁣^*∗∗*^)	0.776 (0.461)
ROCF-delay recall	11.6 (6.22)	12.1 (6.34)	7.85 (5.72)	7.03 (7.04)	8.72 (6.25)	7.37 (5.93)	0.216 (0.642)	10.151 (<0.001⁣^*∗∗*^)	0.627 (0.535)
Attention									
SCWT-C time	45.6 (4.26)	45.3 (4.05)	43.6 (5.63)	42.9 (4.03)	44.3 (5.41)	43.2 (5.66)	0.666 (0.41)	3.866 (0.022⁣^*∗*^)	0.432 (0.649)
TMT-B time	59.2 (25.2)	70.1 (26.7)	77.7 (28.7)	77.5 (26.8)	78.4 (24.9)	91.0 (29.2)	4.819 (0.02⁣^*∗*^)	14.768 (<0.001⁣^*∗∗*^)	0.663 (0.51)
Language									
BNT	21.7 (2.99)	22.8 (2.75)	19.4 (3.94)	19.5 (3.81)	21.0 (3.84)	19.6 (3.40)	0.011 (0.91)	10.611 (<0.001⁣^*∗∗*^)	2.571 (0.079)
VFT	15.9 (3.92)	14.0 (3.31)	13.4 (3.94)	12.7 (3.81)	15.0 (3.50)	12.8 (4.53)	7.834 (0.006⁣^*∗∗*^)	5.728 (0.003⁣^*∗∗*^)	0.398 (0.672)
Attention									
SDMT	36.7 (8.73)	33.2 (10.4)	29.1 (9.69)	25.6 (9.39)	28.5 (12.6)	22.3 (10.2)	8.334 (0.004⁣^*∗∗*^)	21.524 (<0.001⁣^*∗∗*^)	0.791 (0.455)
TMT-A time	48.9 (14.8)	53.8 (25.4)	62.8 (24.3)	64.1 (27.8)	60.4 (24.3)	75.9 (26.9)	4.533 (0.035⁣^*∗*^)	13.118 (<0.001⁣^*∗∗*^)	1.909 (0.151)
Visuospatial skill									
ROCF	26.9 (4.89)	28.1 (4.34)	24.2 (6.65)	22.6 (6.91)	24.8 (6.58)	24.6 (5.51)	0.001 (0.976)	7.408 (<0.001⁣^*∗∗*^)	1.875 (0.173)
CDT	3.72 (0.58)	3.80 (0.50)	3.39 (0.77)	3.40 (0.99)	3.50 (0.66)	3.23 (0.95)	0.152 (0.697)	6.319 (0.002⁣^*∗*^)	1.193 (0.306)

*Note:* The continuous variables in the table are expressed as the mean and standard deviation.

Abbreviations: AVLT, auditory verbal learning test; BNT, Boston naming test; CDT, clock drawing test; EOD, early-onset depression; HC, healthy controls; HighHCY, high homocysteine (homocysteine ≥12.9 μmol/l); LOD, late-onset depression; LowHCY, low homocysteine (homocysteine <12.9 μmol/l); MMSE, mini-mental state examination; ROCF, Rey–Osterrieth complex figure; SCWT, Stroop color word test; SDMT, symbol digit modalities test; TMT, trail making test; VFT, verbal fluency test.

⁣^*∗*^*p* < 0.05, ⁣^*∗∗*^*p* < 0.01.

**Table 3:** The correlation between cognition and plasma homocysteine levels in participants (covariant: age, sex, years of education, HAMD-17 scores, age at first depressive episode, and duration of depression).

Variables	HC (*n* = 89)		EOD (*n* = 57)		LOD (*n* = 56)	
	*r*	*p*	*r*	*p*	*r*	*p*
Global cognition						
MMSE	−0.13	0.237	−0.21	0.144	−0.55	<0.001⁣^*∗∗*^
Memory						
AVLT N1–N5 total	−0.02	0.885	−0.18	0.216	−0.10	0.513
ROCF-delay recall	0.02	0.874	−0.11	0.429	−0.24	0.092
Executive function						
1/SCWT-C time	0.07	0.551	0.10	0.494	−0.30	0.037⁣^*∗*^
1/TMT-B time	−0.10	0.938	−0.21	0.139	−0.24	0.088
Attention						
SMDT	0.09	0.431	−0.27	0.058	−0.30	0.034⁣^*∗*^
1/TMT-A time	0.04	0.693	−0.15	0.305	−0.25	0.080
Language						
BNT	0.10	0.384	0.06	0.691	−0.09	0.519
VFT	−0.07	0.518	0.06	0.656	−0.23	0.103
Visuospatial skill						
ROCF	0.01	0.910	−0.18	0.205	−0.33	0.020⁣^*∗*^
CDT	0.01	0.942	−0.05	0.704	−0.34	0.017⁣^*∗*^

Abbreviations: AVLT, auditory verbal learning test; BNT, Boston naming test; CDT, clock drawing test; EOD, early-onset depression; LOD, late-onset depression; MMSE, mini-mental state examination; ROCF, Rey‒Osterrieth complex figure; SCWT, Stroop color word test; SDMT, symbol digit modalities test; TMT, trail making test; VFT, verbal fluency test.

⁣^*∗*^*p* < 0.05, ⁣^*∗∗*^*p* < 0.01.

## Data Availability

The data that support this study are not publicly available but may be provided upon reasonable request.

## Supporting Information

Supporting Information This document contains additional details regarding Table S1, Table S2, Table S3, and Table S4 that support the findings reported in the main manuscript.

## References

[1] Luppa M., Sikorski C., Luck T. (2012). Age- and Gender-Specific Prevalence of Depression in Latest-Life—Systematic Review and Meta-Analysis. *Journal of Affective Disorders*.

[2] Yeh Y.-C., Tsang H.-Y., Lin P.-Y. (2011). Subtypes of Mild Cognitive Impairment among the Elderly With Major Depressive Disorder in Remission. *The American Journal of Geriatric Psychiatry*.

[3] Taylor W. D., Aizenstein H. J., Alexopoulos G. S. (2013). The Vascular Depression Hypothesis: Mechanisms Linking Vascular Disease With Depression. *Molecular Psychiatry*.

[4] Lozupone M., Castellana F., Sardone R. (2023). Late-Onset Depression but Not Early-Onset Depression May Increase the Risk of All-Cause Mortality in Older Age: 8-Year Follow-Up of the Salus in Apulia Study. *Journal of the American Medical Directors Association*.

[5] Luzzi S., Cherubini V., Falsetti L., Viticchi G., Silvestrini M., Toraldo A. (2022). Homocysteine, Cognitive Functions, and Degenerative Dementias: State of the Art. *Biomedicines*.

[6] Smith A. D., Refsum H., Bottiglieri T. (2018). Homocysteine and Dementia: An International Consensus Statement. *Journal of Alzheimer’s Disease*.

[7] Durga J., van Boxtel M. P. J., Schouten E. G. (2007). Effect of 3-Year Folic Acid Supplementation on Cognitive Function in Older Adults in the FACIT Trial: A Randomised, Double Blind, Controlled Trial. *The Lancet*.

[8] Zhou H., Zhong X., Chen B. (2022). Elevated Homocysteine Levels, White Matter Abnormalities and Cognitive Impairment in Patients With Late-Life Depression. *Frontiers in Aging Neuroscience*.

[9] Kim J.-M., Stewart R., Kim S.-W., Yang S.-J., Shin I.-S., Yoon J.-S. (2008). Predictive Value of Folate, Vitamin B_12_ and Homocysteine Levels in Late-Life Depression. *British Journal of Psychiatry*.

[10] Zhou H., Zhong X., Chen B. (2020). Interactive Effects of Elevated Homocysteine and Late-Life Depression on Cognitive Impairment. *Journal of Affective Disorders*.

[11] Folstein M. F., Folstein S. E., McHugh P. R. (1975). “Mini-Mental State”: A Practical Method for Grading the Cognitive State of Patients for the Clinician. *Journal of Psychiatric Research*.

[12] Guo Q., Chuanzhen L. U., Hong Z. (2000). Application of Rey-Osterrieth Complex Figure Test in Chinese Normal Old People. *Chinese Journal of Clinical Psychology*.

[13] Zhao Q., Lv Y., Zhou Y., Hong Z., Guo Q. (2012). Short-Term Delayed Recall of Auditory Verbal Learning Test Is Equivalent to Long-Term Delayed Recall for Identifying Amnestic Mild Cognitive Impairment. *PLoS ONE*.

[14] Lu J., Guo Q. H., Hong Z., Shi W.-X., Lv C.-Z. (2006). Trail Making Test Used by Chinese Elderly Patients With Mild Cognitive Impairment and Mild Alzheimer’ Dementia. *Chinese Journal of Clinical Psychology*.

[15] Scarpina F., Tagini S. (2017). The Stroop Color and Word Test. *Frontiers in Psychology*.

[16] Nutter-Upham K. E., Saykin A. J., Rabin L. A. (2008). Verbal Fluency Performance in Amnestic MCI and Older Adults With Cognitive Complaints. *Archives of Clinical Neuropsychology*.

[17] Guo Q., Hong Z., Shi W. (1991). Boston Naming Test in Chinese Elderly, Patient With Mild Cognitive Impairment and Alzheimer Dementia. *Chinese Mental Health Journal*.

[18] Sheridan L. K., Fitzgerald H. E., Adams K. M. (2006). Normative Symbol Digit Modalities Test Performance in a Community-Based Sample. *Archives of Clinical Neuropsychology*.

[19] Sunderland T., Hill J. L., Mellow A. M. (1989). Clock Drawing in Alzheimer’s Disease. A Novel Measure of Dementia Severity. *Journal of the American Geriatrics Society*.

[20] Prins N. D., Scheltens P. (2015). White Matter Hyperintensities, Cognitive Impairment and Dementia: An Update. *Nature Reviews Neurology*.

[21] Papazacharias A., Logroscino G., Barulli M. R., Nardini M. (2010). Late Life Depression and Late Onset Depression: Are the Same Clinical and Pathopsysiological Picture?. *Psychiatria Danubina*.

[22] Kamat P. K., Vacek J. C., Kalani A., Tyagi N. (2015). Homocysteine Induced Cerebrovascular Dysfunction: A Link to Alzheimer’s Disease Etiology. *The Open Neurology Journal*.

[23] Shea T. B., Lyons-Weiler J., Rogers E. (2002). Homocysteine, Folate Deprivation and Alzheimer Neuropathology. *Journal of Alzheimer’s Disease*.

[24] Irizarry M. C., Gurol M. E., Raju S. (2005). Association of Homocysteine With Plasma Amyloid *β* Protein in Aging and Neurodegenerative Disease. *Neurology*.

[25] White A. R., Huang X., Jobling M. F. (2001). Homocysteine Potentiates Copper- and Amyloid Beta Peptide-Mediated Toxicity in Primary Neuronal Cultures: Possible Risk Factors in the Alzheimer’s-Type Neurodegenerative Pathways. *Journal of Neurochemistry*.

[26] Li J.-G., Chu J., Barrero C., Merali S., Praticò D. (2014). Homocysteine Exacerbates *β*-Amyloid Pathology, Tau Pathology, and Cognitive Deficit in a Mouse Model of Alzheimer Disease With Plaques and Tangles. *Annals of Neurology*.

[27] Lauriola M., D’Onofrio G., Ciccone F. (2021). Relationship of Homocysteine Plasma Levels With Mild Cognitive Impairment, Alzheimer’s Disease, Vascular Dementia, Psychobehavioral, and Functional Complications. *Journal of Alzheimer’s Disease*.

[28] Den Heijer T., Vermeer S. E., Clarke R. (2003). Homocysteine and Brain Atrophy on MRI of Non-Demented Elderly. *Brain: A Journal of Neurology*.

[29] Williams J. H., Pereira E. A. C., Budge M. M., Bradley K. M. (2002). Minimal Hippocampal Width Relates to Plasma Homocysteine in Community-Dwelling Older People. *Age and Ageing*.

[30] Budge M., Johnston C., Hogervorst E. (2000). Plasma Total Homocysteine and Cognitive Performance in a Volunteer Elderly Population. *Annals of the New York Academy of Sciences*.

[31] Clark M. S., Guthrie J. R., Dennerstein L. (2005). Hyperhomocysteinemia Is Associated With Lower Performance on Memory Tasks in Post-Menopausal Women. *Dementia and Geriatric Cognitive Disorders*.

[32] Li Y.-F. (2020). A Hypothesis of Monoamine (5-HT)—Glutamate/GABA Long Neural Circuit: Aiming for Fast-Onset Antidepressant Discovery. *Pharmacology & Therapeutics*.

