# Supplementary material for: Disparities in Plasma Homocysteine Levels Between Early-Onset and Late-Onset Depression
Source: Depress Anxiety. 2024 Sep 20;2024:7919736. doi: 10.1155/2024/7919736 (PMC11919066; doi:10.1155/2024/7919736)
Supplement: Supporting Information — This document contains additional details regarding Table S1, Table S2, Table S3, and Table S4 that support the findings reported in the main manuscript. [file 7919736.f1.docx]

**Supplementary materials**

Contents

[Table S1. Demographic data, cognitive function, and plasma homocysteine levels of participants using Kruskal-Wallis (nonparametric test). 2](#_Toc164872655)

[Table S2 Depression and cognitive performance of different homocysteine levels in patients using the Mann-Whitney U test (nonparametric test). 4](#_Toc164872656)

[Table S3 Regression weights of the general linear modelfor different homocysteine levels in all participants. (Covariant: age) 7](#_Toc164872657)

[Table S4 The Spearmancorrelation between cognition and plasma homocysteine levels (nonparametric test). 13](#_Toc164872658)

# Table S1.Demographic data, cognitive function, and plasma homocysteine levels of participantsusing Kruskal-Wallis (nonparametric test).

|  | **HC**  ***N=89*** | **EOD**  ***N=57*** | **LOD**  ***N=57*** | **p.overall** | **p.HC vs. EOD** | **p.HC vs. LOD** | **p.EOD vs. LOD** |
| --- | --- | --- | --- | --- | --- | --- | --- |
| Age(years) | 65.0 [63.0;69.0] | 63.0 [61.0;68.0] | 68.0 [65.0;75.0] | <0.001^∗∗^ | 0.034 | <0.001^∗∗^ | <0.001^∗∗^ |
| Years of education | 10.0 [9.00;12.0] | 9.00 [8.00;11.0] | 9.00 [7.00;12.0] | 0.277 | 0.278 | 0.642 | 0.608 |
| Age of first depressive episode (years) | - | 52.0 [46.0;57.0] | 66.0 [63.0;70.2] | <0.001^∗∗^ | - | - | <0.001^∗∗^ |
| Duration of depression (years) | - | 7.00 [3.00;15.0] | 2.00 [1.00;3.25] | <0.001^∗∗^ | - | - | <0.001^∗∗^ |
| HAMD-17 | 0.00 [0.00;2.00] | 9.00 [3.00;15.0] | 10.0 [4.00;17.0] | <0.001 | <0.001^∗∗^ | <0.001^∗∗^ | 0.508 |
| Homocysteine (μmol/L) | 10.9 [9.10;13.0] | 11.3 [9.30;13.0] | 13.1 [10.7;15.3] | 0.002^∗^ | 0.846 | 0.004^∗^ | 0.007^∗^ |
| **Global cognition** | | | | | | | |
| MMSE | 28.0 [27.0;29.0] | 26.0 [23.0;27.0] | 25.0 [22.0;27.0] | <0.001 | <0.001^∗∗^ | <0.001 | 0.264 |
| **Memory** | | | | | | | |
| AVLT N1-N5 total | 34.0 [27.0;39.0] | 27.0 [17.0;34.0] | 27.0 [18.5;31.0] | <0.001 | <0.001^∗∗^ | <0.001^∗∗^ | 0.934 |
| ROCF-Delay Recall | 11.5 [7.00;15.5] | 6.50 [2.88;12.5] | 7.50 [2.50;11.5] | <0.001^∗∗^ | 0.001^∗∗^ | 0.002^∗^ | 0.659 |
| **Executive function** | | | | | | | |
| SCWT-C time | 47.0 [43.0;48.0] | 45.0 [40.0;47.2] | 45.0 [41.0;48.0] | 0.022^∗^ | 0.032^∗^ | 0.080^∗^ | 0.638 |
| TMT-B time | 55.0 [46.0;73.0] | 70.0 [57.2;95.5] | 79.0 [64.0;97.0] | <0.001^∗∗^ | <0.001^∗∗^ | <0.001^∗∗^ | 0.175 |
| **Language** | | | | | | | |
| BNT | 22.0 [20.0;24.0] | 20.0 [17.0;22.0] | 20.0 [18.0;22.0] | <0.001^∗∗^ | <0.001^∗∗^ | 0.007^∗^ | 0.465 |
| VFT | 15.0 [13.0;18.0] | 12.0 [10.0;16.0] | 13.0 [11.0;17.0] | 0.006^∗^ | 0.007^∗^ | 0.052 | 0.444 |
| **Attention** | | | | | | | |
| SDMT | 36.0 [30.0;42.0] | 29.0 [21.5;36.0] | 26.0 [19.0;32.0] | <0.001^∗∗^ | <0.001^∗∗^ | <0.001^∗∗^ | 0.139 |
| TMT-A time | 48.0 [37.0;57.0] | 58.0 [45.0;72.0] | 63.0 [50.0;78.5] | <0.001^∗∗^ | 0.001^∗∗^ | <0.001^∗∗^ | 0.191 |
| **Visuospatial skill** | | | | | | | |
| ROCF | 28.0 [26.0;30.0] | 25.0 [21.5;28.0] | 26.0 [22.8;28.0] | <0.001^∗∗^ | 0.001^∗∗^ | 0.006^∗^ | 0.574 |
| CDT | 4.00 [4.00;4.00] | 4.00 [3.00;4.00] | 4.00 [3.00;4.00] | 0.002^∗^ | 0.008^∗^ | 0.005^∗^ | 0.771 |

The continuous variables presented in the table are median and interquartile range. Abbreviations: HC = healthy controls, EOD = early-onset depression, LOD = late-onset depression, HAMD-17 = Hamilton Depression Rating Scale, 17 items; MMSE = Mini-Mental State Examination; AVLT = Auditory Verbal Learning Test; ROCF = Rey-Osterrieth Complex Figure; SCWT = Stroop Color Word Test; TMT = Trail Making Test; SDMT = Symbol Digit Modalities Test; BNT = Boston Naming Test; VFT = Verbal Fluency Test; CDT = Cand lock Drawing Test.∗p < 0.05, ∗∗p < 0.01.

# Table S2Depression and cognitive performance of different homocysteine levels in patientsusing the Mann-Whitney U test (nonparametric test).

|  | **HC** | | | **EOD** | | | **LOD** | | |
| --- | --- | --- | --- | --- | --- | --- | --- | --- | --- |
|  | **LowHCY** | **HighHCY** | **p.overall** | **LowHCY** | **HighHCY** | **p.overall** | **LowHCY** | **HighHCY** | **p.overall** |
|  | ***N=64*** | ***N=25*** |  | ***N=42*** | ***N=15*** |  | ***N=25*** | ***N=31*** |  |
| Age of first depressive episode (years) | . | . | . | 52.0 [46.2;57.0] | 52.0 [44.5;56.5] | 0.877 | 65.0 [62.0;68.0] | 67.0 [63.0;73.0] | 0.222 |
| Duration of depression (years) | . | . | . | 8.50 [3.00;15.0] | 3.00 [2.25;10.5] | 0.206 | 3.00 [1.00;5.00] | 2.00 [0.75;2.50] | 0.130 |
| HAMD-17 | 1.00 [0.00;2.00] | 0.00 [0.00;3.00] | 0.434 | 7.00 [3.00;15.0] | 9.00 [4.50;15.0] | 0.690 | 8.00 [2.00;14.0] | 10.0 [5.50;17.0] | 0.346 |
| **Global cognition** | | | | | | | | | |
| MMSE | 28.0 [27.0;29.0] | 28.0 [27.0;29.0] | 0.572 | 26.0 [24.0;27.0] | 24.0 [20.5;27.0] | 0.254 | 25.0 [24.0;28.0] | 24.0 [21.5;25.5] | 0.024^∗^ |
| **Memory** | | | | | | | | | |
| AVLT N1-N5 total | 34.5 [30.0;39.0] | 32.0 [24.0;38.0] | 0.117 | 29.5 [18.0;36.5] | 21.0 [16.0;26.5] | 0.099 | 28.5 [21.5;33.8] | 23.0 [18.0;30.0] | 0.171 |
| ROCF-Delay Recall | 11.5 [7.00;15.1] | 11.5 [8.00;17.0] | 0.756 | 7.00 [3.50;12.5] | 6.00 [0.50;10.5] | 0.529 | 8.00 [2.50;12.2] | 7.50 [1.75;10.0] | 0.435 |
| **Executive function** | | | | | | | | | |
| SCWT | 47.0 [44.0;48.0] | 47.0 [43.0;48.0] | 0.734 | 45.0 [42.0;48.0] | 41.0 [40.0;45.5] | 0.348 | 45.5 [41.8;49.0] | 45.0 [40.8;47.0] | 0.352 |
| TMT-B time | 53.5 [42.8;66.5] | 63.0 [52.0;82.0] | 0.034^∗^ | 70.0 [55.0;100] | 70.0 [63.0;84.0] | 0.960 | 73.5 [59.8;92.0] | 93.0 [69.0;104] | 0.067 |
| **Language** | | | | | | | | | |
| BNT | 22.0 [20.0;24.0] | 23.0 [21.0;25.0] | 0.120 | 20.0 [17.0;22.0] | 20.0 [18.0;21.0] | 0.777 | 20.5 [18.0;23.2] | 20.0 [17.0;22.0] | 0.229 |
| VFT | 16.0 [13.0;18.0] | 15.0 [12.0;16.0] | 0.059 | 12.0 [11.0;16.0] | 13.0 [9.50;16.0] | 0.604 | 14.0 [12.0;18.0] | 13.0 [9.50;15.5] | 0.057 |
| **Attention** | | | | | | | | | |
| SDMT | 37.5 [32.0;43.2] | 34.0 [23.0;38.0] | 0.106 | 30.0 [22.5;37.0] | 23.0 [20.5;28.5] | 0.208 | 26.0 [20.8;35.5] | 22.0 [16.0;30.0] | 0.114 |
| TMT-A time | 47.5 [36.8;57.0] | 48.0 [39.0;65.0] | 0.615 | 58.0 [45.0;72.0] | 57.0 [46.5;71.0] | 0.897 | 57.0 [49.2;67.5] | 73.0 [59.5;93.0] | 0.018^∗^ |
| **Visuospatial skill** | | | | | | | | | |
| ROCF | 27.0 [26.0;30.0] | 29.0 [27.0;31.0] | 0.122 | 26.0 [22.5;28.0] | 24.0 [20.0;26.5] | 0.213 | 26.5 [23.8;28.0] | 26.0 [22.2;28.5] | 0.769 |
| CDT | 4.00 [4.00;4.00] | 4.00 [4.00;4.00] | 0.469 | 4.00 [3.00;4.00] | 4.00 [3.00;4.00] | 0.683 | 4.00 [3.00;4.00] | 3.50 [3.00;4.00] | 0.388 |

The continuous variables presented in the table are median and interquartile range.Abbreviations: HC = healthy controls, EOD = early-onset depression, LOD = late-onset depression, LowHCY: low homocysteine (Homocysteine<12.9 μmol/L),HighHCY: High homocysteine (Homocysteine≥12.9 μmol/L),MMSE = Mini-Mental State Examination, AVLT = Auditory Verbal Learning Test, ROCF = Rey-Osterrieth Complex Figure, SCWT = Stroop Color Word Test, TMT = Trail Making Test, SDMT = Symbol Digit Modalities Test, BNT = Boston Naming Test, VFT = Verbal Fluency Test, CDT = Clock Drawing Test.∗p < 0.05, ∗∗p < 0.01.

# Table S3 Regression weights of the general linear modelfor different homocysteine levels in all participants. (Covariant: age)

| Variable | R_squared |  | Estimate | Std_Error | t_value | p_value |
| --- | --- | --- | --- | --- | --- | --- |
| Age of first depressive episode (years) | 0.573 | Intercept | 31.658 | 8.816 | 3.591 | <0.001∗∗ |
|  |  | LOD | 16.289 | 2.213 | 7.362 | <0.001∗∗ |
|  |  | HighHCY | 0.273 | 2.530 | 0.108 | 0.914 |
|  |  | Age | 0.267 | 0.135 | 1.981 | 0.050∗ |
|  |  | LOD*HighHCY | 1.486 | 3.404 | 0.437 | 0.663 |
| Duration of depression (years)P | 0.331 | Intercept | -18.292 | 7.174 | -2.550 | 0.012 |
|  |  | LOD | -10.037 | 1.800 | -5.575 | <0.001∗∗ |
|  |  | HighHCY | -3.883 | 2.059 | -1.886 | 0.062 |
|  |  | Age | 0.456 | 0.110 | 4.159 | <0.001∗∗ |
|  |  | LOD*HighHCY | 2.228 | 2.770 | 0.804 | 0.423 |
| HAMD | 0.343 | Intercept | 5.187 | 5.420 | 0.957 | 0.340 |
|  |  | EOD | 8.447 | 1.291 | 6.541 | <0.001∗∗ |
|  |  | LOD | 8.864 | 1.572 | 5.640 | <0.001∗∗ |
|  |  | HighHCY | 0.020 | 1.584 | 0.013 | 0.990 |
|  |  | Age | -0.056 | 0.082 | -0.682 | 0.496 |
|  |  | EOD*HighHCY | 0.646 | 2.517 | 0.257 | 0.798 |
|  |  | LOD*HighHCY | 1.837 | 2.336 | 0.786 | 0.433 |
| MMSE | 0.305 | Intercept | 29.849 | 2.425 | 12.309 | <0.001∗∗ |
|  |  | EOD | -2.720 | 0.578 | -4.707 | <0.001∗∗ |
|  |  | LOD | -2.668 | 0.703 | -3.794 | <0.001∗∗ |
|  |  | HighHCY | -0.048 | 0.709 | -0.068 | 0.946 |
|  |  | Age | -0.030 | 0.037 | -0.823 | 0.411 |
|  |  | EOD*HighHCY | -1.652 | 1.126 | -1.467 | 0.144 |
|  |  | LOD*HighHCY | -1.970 | 1.045 | -1.884 | 0.061 |
| AVLT N1-N5 total | 0.201 | Intercept | 57.283 | 8.014 | 7.148 | <0.001∗∗ |
|  |  | EOD | -6.821 | 1.852 | -3.682 | <0.001∗∗ |
|  |  | LOD | -4.679 | 2.301 | -2.034 | 0.043∗ |
|  |  | HighHCY | -1.474 | 2.277 | -0.648 | 0.518 |
|  |  | Age | -0.352 | 0.122 | -2.889 | 0.004∗ |
|  |  | EOD*HighHCY | -4.485 | 3.613 | -1.241 | 0.216 |
|  |  | LOD*HighHCY | -2.104 | 3.454 | -0.609 | 0.543 |
| ROCF-Delay Recall | 0.115 | Intercept | 21.223 | 5.436 | 3.904 | <0.001∗∗ |
|  |  | EOD | -3.917 | 1.228 | -3.190 | 0.002∗ |
|  |  | LOD | -2.227 | 1.542 | -1.444 | 0.150 |
|  |  | HighHCY | 1.122 | 1.500 | 0.748 | 0.455 |
|  |  | Age | -0.147 | 0.083 | -1.779 | 0.077 |
|  |  | EOD*HighHCY | -1.878 | 2.376 | -0.791 | 0.430 |
|  |  | LOD*HighHCY | -2.397 | 2.290 | -1.047 | 0.297 |
| SCWT-C time | 0.075 | Intercept | 56.020 | 4.198 | 13.345 | <0.001∗∗ |
|  |  | EOD | -1.995 | 0.963 | -2.073 | 0.040∗ |
|  |  | LOD | -0.552 | 1.188 | -0.464 | 0.643 |
|  |  | HighHCY | 0.584 | 1.198 | 0.487 | 0.627 |
|  |  | Age | -0.160 | 0.064 | -2.513 | 0.013∗ |
|  |  | EOD*HighHCY | -1.296 | 1.883 | -0.688 | 0.492 |
|  |  | LOD*HighHCY | -1.560 | 1.827 | -0.854 | 0.394 |
| TMT-B time | 0.245 | Intercept | -47.559 | 21.733 | -2.188 | 0.030∗ |
|  |  | EOD | 19.116 | 5.058 | 3.779 | <0.001∗∗ |
|  |  | LOD | 11.750 | 6.237 | 1.884 | 0.061 |
|  |  | HighHCY | 3.001 | 6.172 | 0.486 | 0.627 |
|  |  | Age | 1.639 | 0.330 | 4.963 | <0.001∗∗ |
|  |  | EOD*HighHCY | -3.102 | 10.145 | -0.306 | 0.760 |
|  |  | LOD*HighHCY | 8.400 | 9.365 | 0.897 | 0.371 |
| BNT | 0.127 | Intercept | 25.982 | 2.923 | 8.889 | <0.001∗∗ |
|  |  | EOD | -2.269 | 0.676 | -3.358 | 0.001∗ |
|  |  | LOD | -0.327 | 0.839 | -0.390 | 0.697 |
|  |  | HighHCY | 1.448 | 0.830 | 1.744 | 0.083 |
|  |  | Age | -0.066 | 0.044 | -1.490 | 0.138 |
|  |  | EOD*HighHCY | -1.410 | 1.318 | -1.070 | 0.286 |
|  |  | LOD*HighHCY | -2.851 | 1.260 | -2.263 | 0.025∗ |
| VFT | 0.117 | Intercept | 23.165 | 3.305 | 7.009 | <0.001∗∗ |
|  |  | EOD | -2.474 | 0.764 | -3.239 | 0.001∗ |
|  |  | LOD | -0.387 | 0.949 | -0.408 | 0.684 |
|  |  | HighHCY | -1.357 | 0.939 | -1.445 | 0.150 |
|  |  | Age | -0.112 | 0.050 | -2.234 | 0.027∗ |
|  |  | EOD*HighHCY | 0.661 | 1.490 | 0.444 | 0.658 |
|  |  | LOD*HighHCY | -0.745 | 1.425 | -0.523 | 0.602 |
| SDMT | 0.257 | Intercept | 66.392 | 8.558 | 7.757 | <0.001∗∗ |
|  |  | EOD | -7.951 | 1.947 | -4.083 | <0.001∗∗ |
|  |  | LOD | -6.106 | 2.386 | -2.559 | 0.011∗ |
|  |  | HighHCY | -1.333 | 2.361 | -0.564 | 0.573 |
|  |  | Age | -0.455 | 0.130 | -3.500 | 0.001∗ |
|  |  | EOD*HighHCY | -2.029 | 3.749 | -0.541 | 0.589 |
|  |  | LOD*HighHCY | -4.541 | 3.613 | -1.257 | 0.210 |
| TMT-A time | 0.201 | Intercept | -20.325 | 18.706 | -1.087 | 0.279 |
|  |  | EOD | 14.180 | 4.350 | 3.260 | 0.001∗ |
|  |  | LOD | 6.655 | 5.364 | 1.241 | 0.216 |
|  |  | HighHCY | -0.229 | 5.308 | -0.043 | 0.966 |
|  |  | Age | 1.063 | 0.284 | 3.740 | <0.001∗∗ |
|  |  | EOD*HighHCY | 1.642 | 8.443 | 0.194 | 0.846 |
|  |  | LOD*HighHCY | 14.922 | 8.054 | 1.853 | 0.065 |
| ROCF | 0.091 | Intercept | 34.744 | 4.885 | 7.112 | <0.001∗∗ |
|  |  | EOD | -2.786 | 1.129 | -2.467 | 0.015∗ |
|  |  | LOD | -1.609 | 1.402 | -1.147 | 0.253 |
|  |  | HighHCY | 1.750 | 1.388 | 1.261 | 0.209 |
|  |  | Age | -0.120 | 0.074 | -1.617 | 0.108 |
|  |  | EOD*HighHCY | -3.308 | 2.202 | -1.502 | 0.135 |
|  |  | LOD*HighHCY | -1.882 | 2.106 | -0.894 | 0.373 |
| CDT | 0.0813 | Intercept | 4.582 | 0.619 | 7.409 | <0.001∗∗ |
|  |  | EOD | -0.332 | 0.143 | -2.326 | 0.021∗ |
|  |  | LOD | -0.158 | 0.176 | -0.896 | 0.371 |
|  |  | HighHCY | 0.145 | 0.174 | 0.833 | 0.406 |
|  |  | Age | -0.013 | 0.009 | -1.411 | 0.160 |
|  |  | EOD*HighHCY | -0.137 | 0.277 | -0.493 | 0.622 |
|  |  | LOD*HighHCY | -0.410 | 0.266 | -1.538 | 0.126 |

Abbreviations: EOD = early-onset depression, LOD = late-onset depression, MMSE = Mini-Mental State Examination; AVLT = Auditory Verbal Learning Test; ROCF = Rey-Osterrieth Complex Figure; SCWT = Stroop Color Word Test; TMT = Trail Making Test; SDMT = Symbol Digit Modalities Test; BNT = Boston Naming Test; VFT = Verbal Fluency Test; CDT = Clock Drawing Test.∗p < 0.05, ∗∗p < 0.01.

# Table S4 The Spearmancorrelation between cognition and plasma homocysteine levels (nonparametric test).

|  | HC (n=89) | |  | EOD (n=57) | |  | LOD (n=56) | |
| --- | --- | --- | --- | --- | --- | --- | --- | --- |
|  | r | p |  | r | p |  | r | p |
| **Global cognition** | | | | | | | | |
| MMSE | -0.061 | 0.567 |  | 0.007 | 0.956 |  | -0.350 | 0.008** |
| **Memory** | | | | | | | | |
| AVLT N1-N5 total | -0.185 | 0.082 |  | -0.202 | 0.132 |  | -0.135 | 0.341 |
| ROCF-Delay Recall | 0.012 | 0.909 |  | -0.140 | 0.300 |  | -0.112 | 0.437 |
| **Executive function** | | | | | | | | |
| SCWT-C time | -0.001 | 0.992 |  | -0.037 | 0.783 |  | -0.149 | 0.309 |
| TMT-B time | 0.120 | 0.259 |  | 0.039 | 0.778 |  | 0.264 | 0.061 |
| **Attention** | | | | | | | | |
| SMDT | -0.101 | 0.345 |  | -0.185 | 0.176 |  | -0.216 | 0.135 |
| TMT-A time | -0.064 | 0.547 |  | 0.051 | 0.706 |  | 0.266 | 0.059 |
| **Language** | | | | | | | | |
| BNT | 0.057 | 0.598 |  | 0.057 | 0.669 |  | -0.106 | 0.458 |
| VFT | -0.193 | 0.069 |  | 0.011 | 0.932 |  | -0.231 | 0.101 |
| **Visuospatial skill** | | | | | | | | |
| ROCF | 0.092 | 0.386 |  | -0.128 | 0.340 |  | -0.086 | 0.544 |
| CDT | 0.043 | 0.692 |  | 0.202 | 0.134 |  | -0.111 | 0.443 |

Abbreviations: EOD = early-onset depression, LOD = late-onset depression, MMSE = Mini-Mental State Examination; AVLT = Auditory Verbal Learning Test; ROCF = Rey-Osterrieth Complex Figure; SCWT = Stroop Color Word Test; TMT = Trail Making Test; SDMT = Symbol Digit Modalities Test; BNT = Boston Naming Test; VFT = Verbal Fluency Test; CDT = Clock Drawing Test.∗p < 0.05, ∗∗p < 0.01.
